# Comparative Evaluation of Wet and Dry Swallowing in Assessing Eustachian Tube Function in Tympanic Membrane Perforation

**DOI:** 10.1055/s-0045-1811253

**Published:** 2025-10-16

**Authors:** Suresh Kruthika, Jeevan Govindaraju, Hemanth N. Shetty

**Affiliations:** 1 JSS Institute of Speech and Hearing, Mysuru, Karnataka, India

**Keywords:** auditory tube, pressure change, perforation, tympanic membrane, ear infection

## Abstract

**Introduction:**

The Eustachian tube is a pressure-equalizing structure, also called the auditory tube, connecting the middle ear to the nasopharynx. Eustachian tube dysfunction is a leading cause of middle-ear infections leading to tympanic-membrane perforation. Therefore, it is necessary to assess the Eustachian tube functioning before determining the management to avoid recurrent middle-ear infections.

**Objective:**

To compare the functional assessment of the Eustachian tube by measuring pressure changes in patients with tympanic-membrane perforation, during the Toynbee test. The effectiveness of wet versus dry swallowing, ease of swallowing, and test-retest reliability for both conditions were examined.

**Methods:**

The study included 52 ears with tympanic-membrane perforations. The Toynbee test involved applying positive pressure to the ear canal while participants performed dry and wet swallowing maneuvers to measure the resulting pressure changes (in daPa). Subjective difficulty ratings were obtained using a 10-point Likert-scale for both swallowing conditions. Additionally, test-retest reliability for each swallowing condition was evaluated in 20 ears.

**Results:**

The pressure changes observed between the open and closed phases during both swallowing conditions differed significantly. Wet swallowing exhibited a larger initial pressure change than dry swallowing ( *p* < 0.001). Participants rated wet swallowing as significantly easier in contrast to dry swallowing. Additionally, the test-retest reliability for wet swallowing was good (intraclass correlation coefficient [ICC] = 0.737–0.876), while it was poor for dry swallowing (ICC = 0.282–0.350).

**Conclusion:**

Wet swallowing reliably assesses the Eustachian tube's function in tympanic-membrane perforation cases to aid in informed decision making on the possible method of tympanoplasty.

## Introduction

The Eustachian tube (ET) role is essential in the auditory system to regulate middle-ear pressure, a prerequisite for efficient sound transmission. Failure of pressure equalization due to auditory tube dysfunction leads to middle-ear infections, 1 potentially leading to tympanic membrane (TM) perforation, causing pain, discharge, and temporary hearing loss. 2 Trauma to the ear and infections are the leading causes of TM perforations, accounting for 29.1% and 70.9%, respectively. 3 Rehabilitating these patients is essential and generally involves surgical procedures, such as tympanoplasty. This procedure is aimed at reconstructing the perforated TM and improving hearing, and it typically uses a graft, such as a fascia taken from the temporalis muscle, to reconstruct the affected TM. 4 If the ET dysfunction persists after the surgery, it may result in recurrent middle-ear infections and re-rupture of the eardrum. 5 Thus, examining the ET before surgical procedure is necessary to decide on different tympanoplasty methods. Surgery is also required to maintain an intact TM, essential for managing chronic suppurative otitis media (CSOM). 6 Balloon and laser Eustachian tuboplasty enhance ET function and reduce symptoms. 7 Assessing the functions of the ET in patients with TM perforation is essential for selecting appropriate surgical options and graft materials to prevent recurrent perforations caused by ET dysfunction. Several methods are available to evaluate ET in TM perforated cases, such as the saccharin test, Toynbee test, inflation-deflation techniques, and pressure chamber assessments. 8 9 10 A review of the literature indicates that evaluating swallowing may serve as a more effective method for assessing ET function, particularly in relation to the regulation of middle-ear pressure, as measured by tympanometry during the comprehensive nine-step inflation and deflation test in individuals who possess an intact tympanic membrane. 11 However, the influence of dry versus wet swallowing procedures on ET function in individuals with TM perforations resulting from middle-ear infections has yet to be investigated. The present study seeks to fill this gap by evaluating the aforementioned factors. a) The distinction in Eustachian tube function between wet and dry swallowing during the Toynbee test in cases of total TM perforation, and b) the test-retest reliability of wet and dry swallowing in the Toynbee test for the evaluation of ET function.

## Methods

### Study Participants

The study included 52 ears from patients aged between 18 and 68 (mean = 37.33 ± 11.79) years. The study group included 28 female and 24 male participants. The study consisted of 24 left and 28 right ears with a history of discharge lasting between 0.21 and 25.75 (mean = 4.73 ± 5.83) years, and recent discharge varied between 0.04 and 25 (mean = 2.01 ± 4.76) years. The inclusion criteria were patients presenting with TM perforations resulting from recurrent middle-ear infections or chronic otitis media. Additionally, eligible participants had to present dry ears during the evaluation process. Exclusion criteria included CSOM patients , as well as those with ossicular chain disruption, tympanosclerosis, middle-ear atelectasis or neoplasms, active ear discharge, and neuro-motor dysfunctions such as dysarthria, apraxia, and cognitive impairments.

### The Procedure of ET Function Test in Perforated TM

The ET function was assessed using the Clarinet Plus tympanometer (Inventis SRL). This allowed for controlled pressure application and precise measurement of middle-ear pressure changes during the Toynbee test. The ET function was examined through a sequence of pressure equalization test designed to assess the auditory tube's ability to open and equalize the pressure of the middle-ear cavity. For this purpose, dry and wet swallowing maneuvers were included in the Toynbee test. The patient received explicit instructions regarding the swallowing procedures for each maneuver and was advised to maintain a comfortable seated position throughout the duration of the testing process. For the Toynbee test, a tympanometry probe tip was placed in the patient's ear, and an initial positive pressure of +300 daPa was applied to the ear canal. This pressure created a difference between the middle ear and the outside environment. Once the air pressure was applied, patients were instructed to perform dry or wet swallowing maneuvers. These maneuvers help open the ET and allow ventilation between the middle ear and the nasopharynx. Each time the patient swallowed, the tympanometer recorded the change in air pressure of the middle-ear cavity. Applying pressure to carry out swallowing maneuvers was repeated multiple times to assess the consistency and effectiveness of the pressure equalization with each swallowing maneuver. The tympanometer adjusted the pressure gradually, both in positive and negative directions, in a “staircase” manner, to thoroughly assess how well the ET responded to the varying pressures and whether it could equalize pressure effectively.

**Fig. 1:**
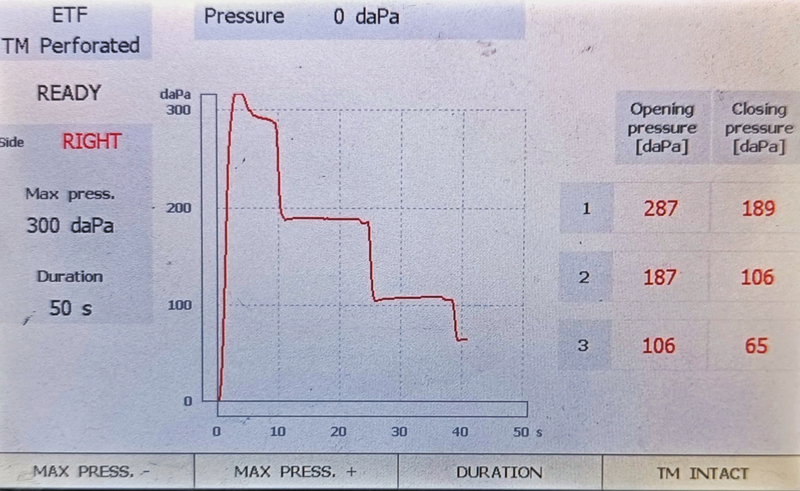
Representative tympanometry tracings of pressure (in daPa) for the opening and closing phase in dry swallowing condition.

The middle-ear pressure would decrease with each swallow as the ET opened, allowing excess middle-ear pressure to be equalized to atmospheric pressure. Every time the swallowing meneuver was performed, this pressure drop was noted. Both dry and wet swallowing were conducted to evaluate the functioning of the ET. Participants performed dry and wet swallowing maneuvers randomly, which were counterbalanced among participants to measure the pressure (in daPa). A total of 26 ears were tested first under dry swallow conditions, followed by testing with wet swallowing. The other 26 ears were tested in the opposite order. During the wet swallowing condition, water was provided to the patient in a bottle, and a straw was used to sip the water. This helped reduce head movement, which could interfere with maintaining the hermetic seal necessary for accurate pressure measurements with the tympanometer. Figure 1 provides a representative tympanometry tracing of pressure (in daPa) for the opening and closing phases obtained in the dry swallowing condition.

After completing each swallowing condition, the patients were instructed to rate the difficulty of each swallowing maneuver using a 10-point Likert scale. The instruction was: How would you rate the difficulty of swallowing?. The Likert scale was anchored at 1 with no difficulty with swallowing and 10 with very difficult to swallow. The scale was thoroughly explained to ensure that participants could accurately evaluate their difficulty levels in each condition. 12 To verify the reliability of the test procedure, a test-retest evaluation was performed on a group of 20 participants a few hours later. It was done to evaluate the consistency and reproducibility of the results across multiple sessions, providing additional insight into the reliability of the Toynbee test procedure and the swallowing maneuvers for assessing ET function.

### Statistical Analysis

Data was analyzed using the IBM SPSS Statistics for Windows (IBM Corporation) software, version 21.0. Four ears were excluded from the analysis as they had patent ET, which affected the pressure required for the ET function tests. The Wilcoxon signed-rank test evaluated pressure changes between the open and closed phases for each swallowing condition and compared the pressure changes between wet and dry swallowing conditions. This test also helped us determine which condition (wet or dry) led to a more significant change in pressure. We used the intraclass correlation coefficient (ICC) test to check the reliability of the pressure changes during wet and dry swallowing.

## Results

### Opening vs. Closing Phase

**Fig. 2:**
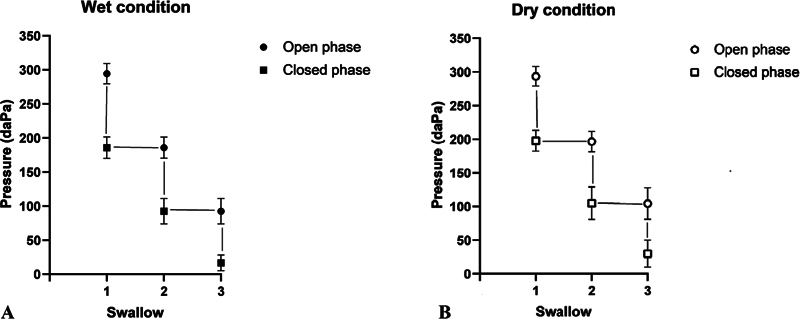
Pressure (in daPa) for the opening and closing phase for wet ( **A** ) and dry ( **B** ) swallowing conditions.

The data on pressure in the opening and closing phases during three swallowing conditions were subjected to a Wilcoxon signed-rank test to determine if there was a change in pressure between the open and closed phases of the Toynbee test. As expected, a lower pressure was observed in the closed phase than in open phase for the wet swallowing condition ( Fig. 2A ). This difference was found significant between the open and closed phases in swallows 1 (mean = 294.46 ± 4.98 daPa of the open phase; mean = 185.833 ± 15.72 daPa of the closed phase; Z = -6.032; *p* < 0.001), 2 (mean = 185.83 ± 15.55 daPa of the open phase; mean = 92.50 ± 18.70 daPa of the closed phase; Z = -6.033; *p* < 0.001), and 3 (mean = 92.42 ± 18.59 daPa of the open phase; mean = 16.71 ± 11.50 daPa of the closed phase; Z = -6.032; *p* < 0.001). Similarly, a lower pressure was noticed in the closed compared with the open phase for the dry swallowing condition ( Fig. 2B ). This difference was found to be significant between the open and closed phases in swallows 1 (mean = 293.67 ± 4.47 daPa of open phase; mean = 196.77 ± 15.48 daPa of the closed phase; Z = -6.032; *p* < 0.001), 2 (mean = 196.67 ± 15.17 daPa of the open phase; mean = 104.67 ± 24.09 daPa of the closed phase; Z = -6.032; *p* < 0.001), and 3 (mean = 104.31 ± 23.59 daPa of the open phase; mean = 29.73 ± 19.96 daPa of the closed phase; Z = -6.032; *p* < 0.001).

### Dry vs Wet Swallowing Condition

**Fig. 3:**
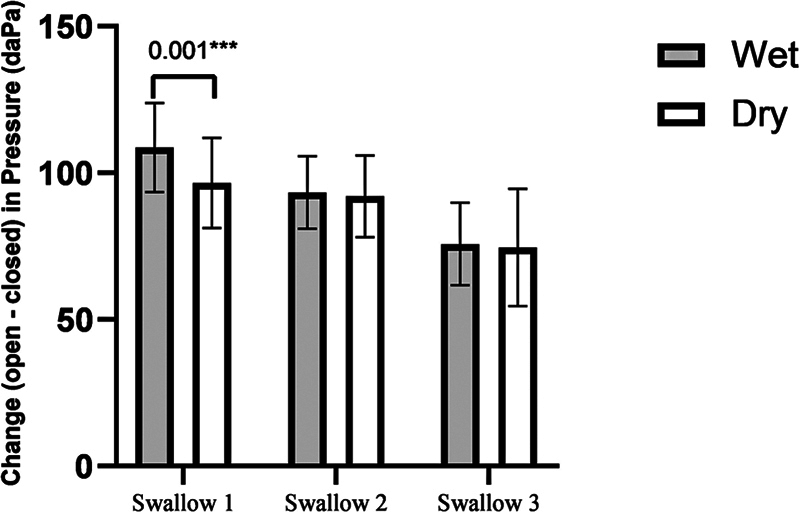
Change in pressure (in daPa) in wet and dry swallowing conditions.

Pressure change is obtained by subtracting the pressure recorded during the open phase from that of the closed phase. The pressure change between the dry and wet swallowing conditions was analyzed using a Wilcoxon signed-rank test. Figure 3 shows the change in pressure (in daPa) in wet and dry swallowing conditions. A more considerable pressure change was observed in wet than dry in swallow one, which was found significant (mean = 108.63 ± 15.17 daPa of pressure change in the wet phase; mean = 96.90 ± 15.36 daPa of pressure change in the dry phase; Z = -5.219; *p* < 0.001). Although pressure change was found to be relatively larger in the wet than in the dry phase, this difference failed to reach significance (mean = 93.33 ± 12.35 daPa of pressure change in the wet phase; mean = 92.00 ± 13.89 daPa of pressure change in the dry phase; Z = -0.445; *p* = 0.530) in swallow 2 and (mean = 75.71 ± 14.05 daPa of pressure change in wet phase; mean = 74.58 ± 16.96 daPa of pressure change in dry phase; Z = -0.796; *p* = 0.562) in swallow 3.

### Subjective Rating on Ease of Swallow

**Fig. 4:**
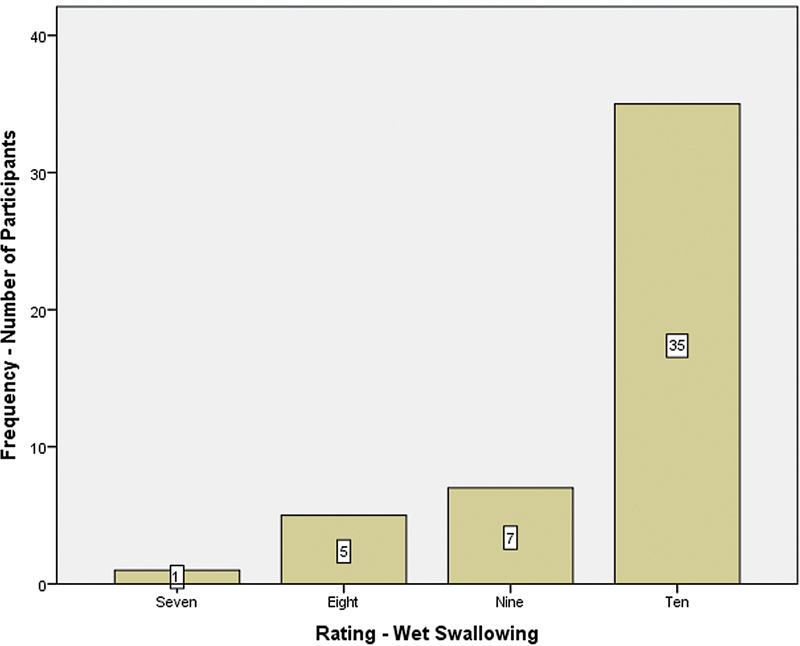
Subjective rating of ease of swallowing on wet swallow condition.

Each participant rated the ease of swallowing in dry and wet swallow conditions on a Likert scale of 1 to 10, according to which 1 indicated the highest difficulty and 10 indicated ease of swallowing. Fig. 4 represents the subjective rating of ease of swallowing on wet swallowing condition. In wet swallowing condition, out of 48 participants, 73% (35 participants) rated 10, followed by 15% (7 participants) who rated it 9, 10% (5 participants) who rated it 8, and only 2% (1 participant) who rated it 7. The rating of ease of swallowing for the wet swallow condition ranged from 10 to 7, and most participants rated it as 10.

**Fig. 5:**
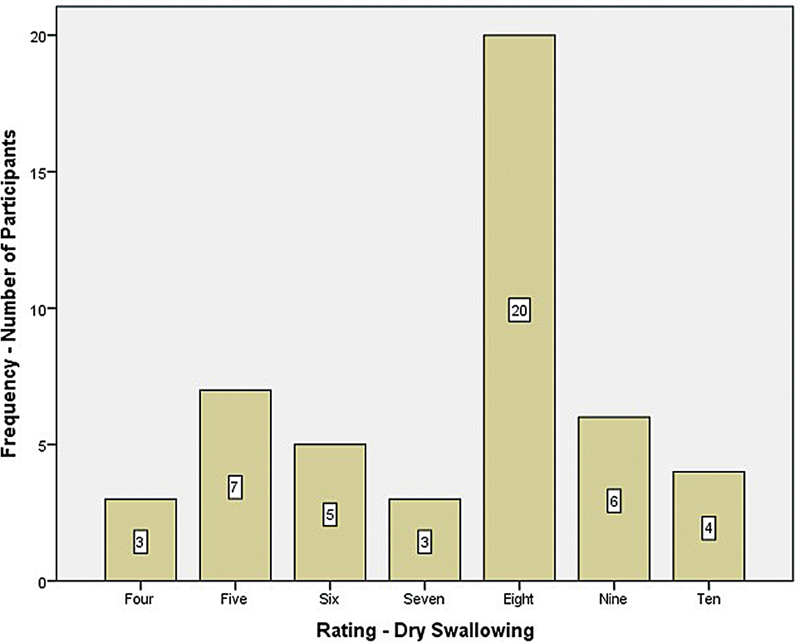
Subjective rating of ease of swallowing on dry swallow condition.

For the dry swallowing condition, 8% (4 participants) rated it 10, followed by 13% (6 participants) who rated it 9, 42% (20 participants) who rated it 8, 6% (3 participants) who rated it 7, 10% (5 participants) who rated it 6, 15% (7 participants) who rated it 5, and 6% (3 participants) who rated it 4 ( Fig. 5 ). The rating of ease of swallowing for the dry swallowing condition ranged from 10 to 4, and most participants rated it 8.

### Reliability of Wet and Dry Swallow Conditions

Reliability was assessed in 42% (20 participants) of the sample on a pressure change in the 3 swallowing conditions in wet and dry conditions using the ICC. In wet swallowing conditions, the reliability of pressure changes in swallows 1 (r = 0.876) and 3 (r = 0.860) yielded good reliability. However, the reliability of pressure change was found to be moderate in swallow 2 (r = 0.737). A similar test was employed to assess reliability in pressure change for the dry swallowing condition, which revealed poor correlation in the 1st (r = 0.350), 2nd swallow (r = 0.282), and 3rd swallows (r = 0.337).

## Discussion

The present study's findings explain how various swallowing conditions affect ET function, especially in individuals with perforated TM due to middle-ear infections. The study revealed significant differences in pressure change between the open and closed phases in wet and dry swallowing conditions, suggesting that the ET opened successfully during the swallowing maneuver to equalize middle-ear pressure. This result ensures that the Toynbee test effectively evaluated the ET function in the context of TM perforations. 13 The mean pressure change during swallowing is highest in the first swallow, followed by the second and third. The first swallow is important for equalizing middle-ear pressure to the atmospheric pressure in the external environment, while later swallows only make minor changes. Muscle adaptation and decreased ET responsiveness diminish the impact of subsequent swallows, highlighting the importance of the initial swallow. Also, the comparison of pressure changes between wet and dry swallowing conditions indicated a significant pressure change in wet swallowing in the first swallow. The second and third swallows, however, revealed no significant variations. Wet swallowing has a more significant initial pressure change than dry swallowing because it activates the muscles of the ET more intensely, which are required for opening the ET. Additionally, the lubricating properties of the water used in wet swallowing help the surrounding soft tissues move more smoothly and enhance the ET's capacity to control pressure during the early swallowing process. The results of the present study align with those found using sonotubometry to assess ET function, in which a higher intensity (measured in sound pressure level) is observed in the ear canal in wet swallowing compared to dry swallowing condition in individuals with ET dysfunction or TM perforation. 14 15 16 The reason could be wet swallowing aids ET opening in cases with TM perforation due to ET dysfunction by providing lubrication, reducing tissue resistance, and exerting mechanical pressure on the ET orifice, compensating for the altered biomechanics and facilitating better ventilation.

In the current study, the subjective ratings of ease of swallowing revealed that most participants found wet swallowing significantly easier than dry swallowing. About 73% of participants found wet swallowing very easy, while only 8% felt the same about dry swallowing, which caused discomfort during the test. The ease of swallowing greatly enhanced pressure equalization, as shown by a significant increase in pressure change during the initial swallowing phase. These results highlight the role of patient comfort in evaluating ET function. When patients find the test easier to perform, it positively influences the accuracy and reliability of the test results. Comparable to the tympanometry technique used to evaluate ET function in cases with TM perforation, another method mentioned in the literature is sonotubometry. A notable advantage of sonotubometry is the absence of pressurization in the external auditory canal, which may enhance comfort and compliance, particularly among the pediatric and postoperative populations. However, the sonotubometry method is susceptible to external and physiological internal noises. It requires high patient cooperation, making the study method more desirable for clinical populations to obtain valid results. 17 18 The test-retest reliability assessment of tympanometry was used to assess the ET function in TM perforated cases, which showed better consistency in pressure changes during wet swallowing than in dry swallowing conditions. These findings indicate that wet swallowing could be a more reliable way of assessing ET function in patients with perforated TM.

## Conclusion

The Toynbee test evaluates ET function in patients with perforated TM. It was found that wet swallowing yields a larger pressure change than dry swallowing, as the liquid facilitates the movement of soft-palate muscles to open the ET. Many patients reported that wet swallowing is easier, enhancing the accuracy of results than dry swallowing. Consistent pressure changes were observed during wet swallowing, indicating its effectiveness. This study emphasizes the functional assessments of the ET in cases with TM perforation and aids in making informed decisions regarding surgical rehabilitation.
